# Effect of Hypoxia on Adult Müller Glia Cultures

**DOI:** 10.3390/biomedicines13071743

**Published:** 2025-07-16

**Authors:** Xabier Miguel-López, Laura Prieto-López, Elena Vecino, Xandra Pereiro

**Affiliations:** 1 Experimental Ophthalmo-Biology Group, Department of Cell Biology and Histology, University of Basque Country UPV/EHU, 48940 Leioa, Spain; xmiguellopez@gmail.com (X.M.-L.); laura.prieto@ehu.eus (L.P.-L.); 2 Begiker-Ophthalmology Research Group, BioCruces Health Research Institute, Cruces Hospital, 48903 Barakaldo, Spain

**Keywords:** Müller glia, hypoxia, retina, metabolic stress

## Abstract

**Background**: The retina, a light-sensitive tissue of the central nervous system that is located at the posterior part of the eye, is particularly vulnerable to alterations in oxygen levels. In various retinal diseases, such as central retinal vein occlusion, glaucoma, and diabetic retinopathy, hypoxia (a condition of low oxygen levels) is commonly observed. Müller glia, the principal glial cells in the retina, play a crucial role in supporting the metabolic needs of retinal neurons. They are also responsible for sensing oxygen levels and, in response to hypoxia, express Hypoxia-Inducible Factor 1 (HIF-1), a transcription factor that activates signaling pathways related to hypoxia. **Methods:** In this study, primary rat Müller glial cells were cultured and exposed to a 1% oxygen for 72 h. Following this, immunohistochemical assays were conducted to assess the effects of hypoxia on various parameters, including HIF-1α expression, cell survival, Müller glia-specific markers (CRALBP and GS), gliosis (GFAP expression), apoptosis (caspase-3 expression), cell proliferation (Ki-67 expression), and metabolic stress (indicated by the number of mitochondria per cell). **Results:** Under hypoxic conditions, a decrease in Müller glial survival and proliferation was observed. Conversely, there was an increase in HIF-1α expression, GFAP expression, caspase-3-positive cells, and the number of mitochondria per cell. However, no significant changes were noted in the expression of the Müller glial markers GS and CRALBP. **Conclusions:** In conclusion, hypoxia resulted in reduced proliferation and survival of Müller glial cells, primarily due to increased apoptosis and heightened metabolic stress.

## 1. Introduction

Müller glia (MG) is the predominant non-neuronal cell type in the vertebrate retina and account for up to 90% of retinal glia. These cell bodies exhibit a distinctive radial morphology, extending across the entire thickness of the retina—from the inner to the outer layer—which enables them to interact with all retinal neurons and cell types [1,2]. Due to the close contact with all the cells of this tissue, apart from providing structural stability, MG are involved in a multitude of essential retinal activities, constitute a common link between all retinal cell types, and establish metabolic associations with them, in addition to maintaining homeostasis or guiding light to the photoreceptors [2,3]. It is due to this tight junction that MG are able to transport most of the nutrients, waste products, ions, water, oxygen, and other molecules to the retinal blood vessels and neurons [2].

MG have multiple functions, but one of their main functions is to maintain the blood–retinal barrier, the integrity of which is necessary for proper retinal function. Normally, MG enhance the barrier function of vascular endothelia by secreting many different factors [1,2]. Furthermore, it has been observed that MG may act as a communication system for metabolic exchange between blood vessels and neurons [2]. Beyond their structural role, MG are crucial for neuroprotection and the regulation of synaptic activity. Their neuroprotective functions encompass a variety of mechanisms, including the uptake of excess glutamate, which is neurotoxic to retinal ganglion cells (RGCs). Additionally, MG are involved in maintaining and recycling neurotransmitters that are essential for nerve signaling and supplying neurotransmitter precursors. They also play a key role in regulating glucose and glycogen metabolism by providing neurons with vital nutrients such as lactate and pyruvate [2,3]. Neurotrophic factors released by MG are involved in the regulation of retinal neuronal circuits and in the protection of RGCs. Likewise, many neuroprotective factors have been identified that increase the survival of both photoreceptors and RGCs [2,4,5,6].

The retina has metabolic peculiarities related to its activity. Glucose from internal cellular glycogen stores or lactate is the primary metabolite used by retinal cells to produce energy, both under aerobic and anaerobic conditions. The glial cells and neurons carry out complex metabolic relationships that adjust their metabolic activity to their needs. For example, MG are able to resist early stages of hypoxia and low-glucose environments by activating anaerobic glycolysis and oxidating alternative substrates, such as lactate, glutamate, or glutamine, to obtain energy in the form of ATP [2,7].

The retina has one of the highest oxygen consumption rates per tissue volume compared to other organs [8]. Its high energy demand is due to the retina being a highly sensitive and efficient system that converts light energy into neural signals, which is why the retina consumes oxygen more rapidly than other tissues [9,10]. In times of increased energy demand, oxygen becomes one of the most limited metabolites in this tissue. For this reason, the retina is susceptible to alterations in oxygen levels, making it susceptible to hypoxia, that is, a lack of oxygen to an organism, tissue, or cell [11].

These conditions may derive from the cardiovascular effects of chronic obstructive airway disease, ocular ischemic syndrome associated with arterial obstructive conditions such as carotid artery stenosis, hyperviscosity syndromes, or trauma [12]. These pathologies, which cause oxygen deprivation in the retina, can lead to several vision-threatening disorders, such as central retinal vein occlusion [13]. Similarly, pathologies such as diabetic retinopathy, glaucoma, or retinal occlusive vasculitis have also been observed to be affected by hypoxia [12,14]. Furthermore, it has been described that RGCs are particularly sensitive to hypoxic stress, which contributes to their degeneration, leading to irreversible vision loss [12]. Under hypoxic conditions, it is known that MG can be activated rapidly, remove metabolic waste, and maintain the balance of the extracellular environment of the retina to protect RGCs [15]. This activation occurs through the expression of Hypoxia-Inducible Factor (HIF), which is a transcription factor that is responsible for the activation of genes that facilitate the adaptation and survival of cells to hypoxia [16].

Given the crucial role of MG in the neuroprotection of RGCs, which maintain retinal metabolic homeostasis and act as the retina’s primary oxygen sensors, understanding how hypoxia impacts adult MG is of great interest. An in vitro hypoxia model of adult MG will help us explore their responses and potential involvement in the progression of age-associated neovascular retinal diseases. Therefore, in this study, we aim to investigate the effects of hypoxia on adult MG by examining the impact of low oxygen levels on HIF-1α, MG-specific markers, cell survival, apoptosis, gliosis, proliferation, and metabolic stress in adult primary MG cultures.

## 2. Materials and Methods

Two-month-old adult Sprague-Dawley rat eyes (*Rattus norvegicus domestica*) (*n* = 20) were obtained from animals that were housed under a 12 h light–dark cycle with ad libitum access to food and water and humanely sacrificed by exposure to CO_2_. The animal experimentation adhered to the ARVO Statement for the Use of Animals in Ophthalmic and Vision Research. Moreover, all the experimental protocols complied with the European (2010/63/UE) and Spanish (RD53/2013) regulations regarding the protection of experimental animals, and they were approved by the Ethics Committee for Animal Welfare at the University of the Basque Country.

### 2.1. Retina Extraction and Primary Müller Glia Cultures

**Figure 1:**
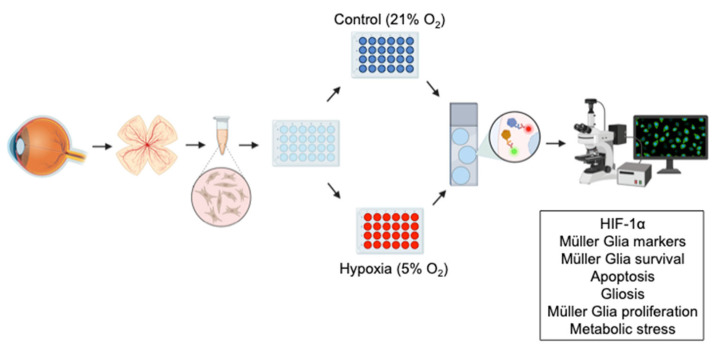
Methodology used in this experiment. Created with www.BioRender.com (accessed on 2 June 2025).

Rat retinas were enucleated and maintained in CO_2_-independent medium (Life Technologies, Carlsbad, CA, USA), the cornea and anterior chamber were separated from the rest of the eye, and both the crystalline and vitreous humor were removed. Next, the retina was isolated from the sclera and cut by the optic nerve. Then, MG were cultured according to the protocols established previously [17,18]. Briefly, the retina was digested at 37 °C for 30 min with papain (20 U/mL; Worthington, Lakewood, NJ, USA) and DNase I (2000 U/mL; Worthington, Lakewood, NJ, USA) in Sterile Earle‘s Balanced Salt Solution (EBSS). This enzymatic digestion was stopped by adding Dulbecco’s Modified Eagle’s Medium (DMEM; Life Technologies, Carlsbad, CA, USA) containing 10% Fetal Bovine Serum (FBS; Life Technologies, Carlsbad, CA, USA), and the retina was dissociated mechanically. The obtained cell homogenate was centrifuged at 1200 rpm 5 min to remove debris, and the pellet was resuspended in DMEM + 10% FBS. Then, 1 × 10^5^ viable cells per well were seeded in poly-l-Lysine (100 μg/mL; Sigma-Aldrich, St. Louis, MO, USA)- and laminin (10 mg/mL; Sigma-Aldrich, St. Louis, MO, USA)-coated 13 mm sterile coverslips on a 24-well plate. The cell cultures were maintained in a humidified incubator at 37 °C in an atmosphere of 5% CO_2_. The medium was changed on day 1 of the culture, and half the volume of the medium was replaced every 2 days until the cultures were in a pre-confluent state. When the primary MG reached this state, their conditions were changed to hypoxic conditions (1% O_2_, 5% CO_2_), whereas the control plate was maintained in 21% O_2_ and 5% CO_2_ for 72 h (Figure 1).

### 2.2. Immunocytochemical Analysis and Image Capture

The cells were washed three times in Phosphate-Buffered Saline (PBS, pH 7.4), fixed in methanol at −20 °C for 10 min, and washed again three times. Later, non-specific antigen binding was blocked with blocking buffer (0.1% Triton X-100 and 3% Bovine Serum Albumin—BSA—in PBS). The primary antibodies (Table 1) were diluted in blocking buffer and incubated with the cells overnight at 4 °C. After washing three times in PBS, cells were incubated for 1 h at room temperature with the corresponding secondary antibodies, which were diluted 1:1000; Alexa Fluor 488-conjugated goat anti-mouse; Alexa Fluor 555-conjugated goat anti-rabbit antibodies (Invitrogen, Carlsbad, CA, USA); Alexa Fluor 488-conjugated goat anti-rabbit; Alexa Fluor 555-conjugated goat anti-mouse antibodies (Invitrogen, Carlsbad, CA, USA); and DAPI 1:10,000. After a further three washes with PBS, the coverslips were mounted with Fluor-save Reagent (Sigma-Aldrich, St. Louis, MO, USA). The MG were analyzed on an epifluorescence microscope (Zeiss, Jena, Germany) coupled to a digital camera (Zeiss Axiocam MRM, Zeiss, Jena, Germany) (Figure 1). Two visualizevimentin organization images were acquired using a high-resolution Leica Stellaris 5 confocal microscope (Leica Microsystems, Wetzlar, Germany) under 63× magnification.

### 2.3. Quantification and Statistical Analysis

For the cell survival quantification, a mosaic of the entire coverslip was obtained with a 10× objective, and once the mosaic was defined, the coverslip surface area was calculated (132.73 mm^2^). The semi-automatic Zen software v. 3.7 (Zeiss) was used to count the total number of nuclei that were stained with DAPI, taking into consideration the limits of the axis of the nuclei of MG to achieve more accurate measurements. As such, we used a specific macro, designed to measure the limits of the axes (10–40 μm), which was corrected manually for each image. Using Image J software (v.1.48), MG that were positive for Caspase 3 and Ki-67 and mitochondria were manually quantified. To analyze the cell area, the cell contours were manually drawn using the Freehand Selections tool in ImageJ (v.1.48), and subsequently the cell area was automatically detected. Measurement of GFAP expression was also performed with Image J software, measuring the integrated density to obtain an accurate quantification of fluorescence, considering the area of the region and minimizing the impact of noise [19]. In the analysis, the survival of the MG under each condition was considered, as it influences the measured fluorescence, allowing for normalization of the integrated density values. Three levels of intensity thresholds (low, medium, and high) were established by analyzing controls for each condition, considering the low-intensity control as the 100%. Three coverslips per condition were analyzed in at least three independent experiments.

Statistical analyses were carried out with IBM SPSS Statistical v.24-0, and the means and standard error of the mean (SEM) for each condition are presented. A Mann–Whitney U test was used to evaluate whether there were significant differences between means. Differences were considered significant for all tests at *p*-value ≤ 0.05.

## 3. Results

### 3.1. MG Markers and Cell Survival Assay

**Figure 2:**
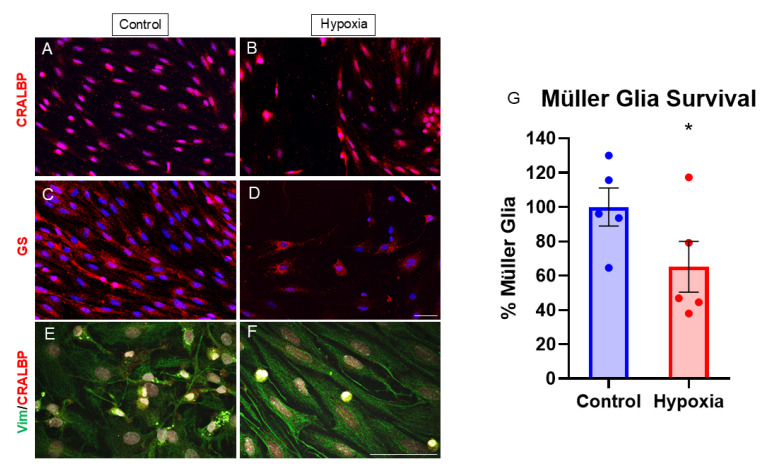
Analysis of survival and expression of CRALBP and GS in MG cultures under control and hypoxia conditions. Images of MG cultures taken under control (**A,C,E**) and hypoxia (**B,D,F**) conditions, in which the expressions of CRALBP (red) (**A,B,E,F**), GS (red) (**C,D**), and Vimentin (**E,F**) are observed. The immunostaining intensities and patterns of CRALBP and GS appeared unchanged when comparing the two conditions. In the analysis of cell survival, a significant decrease in the total number of cells in hypoxia is observed compared to the control (**G**). Changes in vimentin organization as observed by confocal microscopy at 63×. Nuclei were labeled with DAPI (blue). * *p*-value ≤ 0.05. Scale bar = 50 µm.

In order to study how hypoxia affects MG, the expressions of molecular markers of MG were analyzed in rat cultures, specifically the expressions of glutamine synthetase (GS), CRALBP, and vimentin. The expressions of these specific MG markers were evident, showing no differences in the expression of CRALBP (Figure 2A,B,E,F) and GS (Figure 2C,D) in hypoxia compared to control conditions. Nevertheless, the survival of MG was analyzed in control cultures and those subjected to hypoxia. For this purpose, the coverslips were analyzed in their entirety (Supplementary Figure S1), and the percentage of cells in hypoxia compared to the control was calculated. In these, it was observed that by subjecting the cells to 1% O_2_, cell survival in hypoxia compared to the control decreased significantly to 65.17 ± 14.86% (Figure 2G).

Moreover, under low-oxygen conditions, vimentin fibers (an important component of the cytoskeleton) appear to change in their organizational pattern under hypoxic conditions compared to controls. Specifically, in control MG cultures, vimentin fibers display a predominantly parallel, linear organization (Figure 2E). Under hypoxia, vimentin fibers adopt a different arrangement, appearing more reticulated and with less uniform alignment, although the overall vimentin expression levels remain unchanged (Figure 2F).

### 3.2. Expression of HIF-1α

**Figure 3:**
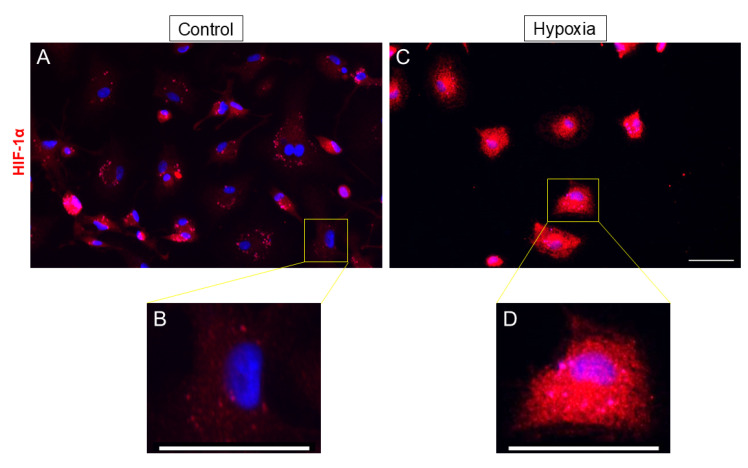
Effect of hypoxia on HIF-1α expression. Images of MG cultures taken under control (**A,B**) and hypoxia (**C,D**) conditions ((**B,D**) images are zoomed-in images of (**A,C**), respectively). It can be observed that under hypoxic conditions, there is a greater expression of HIF-1α marker. Cells were labeled with anti-HIF-1α (red), and the cell nuclei were labeled with DAPI (blue). Scale bar = 50 µm.

Once we had assessed the presence and purity of the MG, we studied the effect of hypoxia by evaluating HIF-1α expression, a cellular transcription factor that is activated by a lack of oxygen. In control cultures, low intensity was found in the cell cytoplasm, apart from some cells that showed a high intensity (Figure 3A). By contrast, in cells that were subjected to hypoxia, the labeling was intense in the cytoplasm of the MG (Figure 3C). Since HIF-1α is a transcription factor, we examined its nuclear localization as an indicator of hypoxic response. Under normoxic conditions, MG cell cultures displayed little to no nuclear labeling. In contrast, exposure to hypoxia resulted in a marked increase in the nuclear HIF-1α signal. These findings indicate that MG cells respond to hypoxic stress by upregulating HIF-1α expression, with prominent accumulation being observed in both the nucleus and cytoplasm.

### 3.3. Hypoxia Mediated-Apoptosis

**Figure 4:**
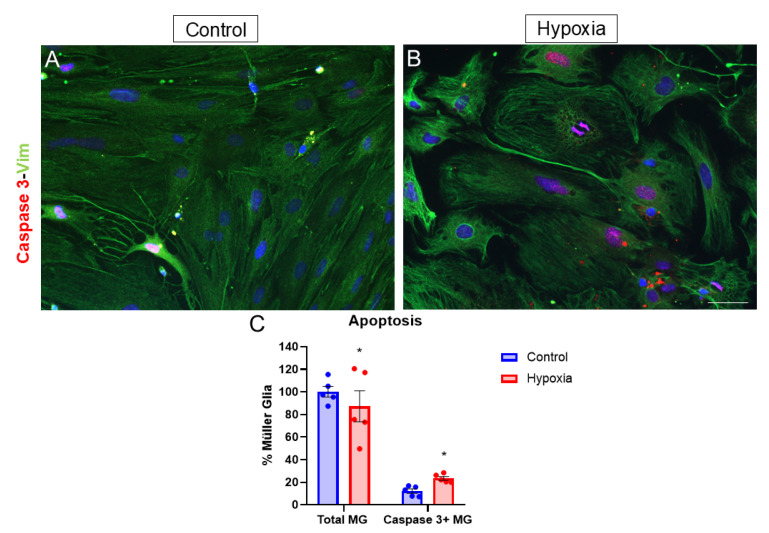
Analysis of apoptosis by expressing caspase 3. Images of MG cultures were taken under control (**A**) and hypoxia (**B**) conditions, in which the expressions of caspase 3 (red) and vimentin (green) are observed. A significant increase in the number of caspase 3-positive cells can be observed under hypoxic conditions compared to the control (**C**). Nuclei were labeled with DAPI (blue). * *p*-value ≤ 0.05. Scale bar = 50 µm.

To study hypoxia-mediated apoptosis, MG that were positive for caspase 3, an apoptosis marker, were quantified in hypoxia compared to control cultures. Both under control and hypoxic conditions, nuclear labeling was observed (Figure 4A,B). Under control conditions, 12.18 ± 1.99% of the cells present were caspase 3, while under hypoxic conditions, the percentage increased significantly to 23.45 ± 1.64% (Figure 4C). In addition, the number of total cells under hypoxic conditions decreased to 87.23% ± 13.7% (Figure 4C).

### 3.4. Gliosis by GFAP Expression

**Figure 5:**
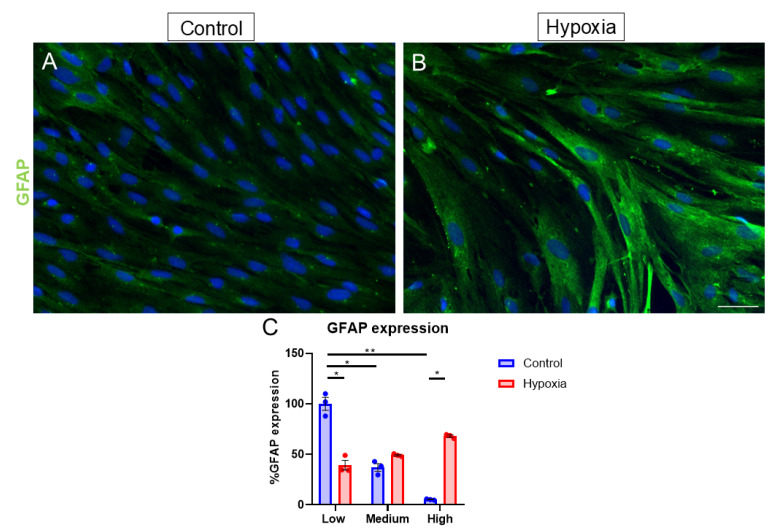
Effect of hypoxia on the expression of the gliosis marker GFAP under control and hypoxia conditions. Images of MG cultures taken under control (**A**) and hypoxia (**B**) conditions, in which the expression of GFAP (green) is observed. A greater expression of GFAP can be observed under hypoxic conditions compared to the control (**C**). Nuclei were labeled with DAPI (blue). * *p*-value ≤ 0.05. ** *p*-value ≤ 0.01. Scale bar = 50 µm.

To determine if hypoxia can induce gliosis in MG, cytoskeletal protein GFAP was studied in the MG cell cultures. A quantitative analysis of the GFAP expression was performed by measuring the fluorescence intensity of GFAP staining in the images. Specifically, we classified the intensity in each cell as low, medium, or high, as higher levels of GFAP expression are considered a hallmark of reactive gliosis in MG. The classification was based on the relative GFAP, considering the low-intensity control as 100% In control cultures (Figure 5A), the GFAP expression decreased to medium and high levels of GFAP (37.08 ± 3.83% and 5.36 ± 0.33%, respectively) (Figure 5C); on the other hand, in hypoxia-exposed cultures (Figure 5B), the GFAP expression levels rose from 39.73 ± 4.84% (low expression) to 49.28 ± 0.76% (medium expression), with its peak being high expression levels at 68.05 ± 1.16% (Figure 5C). Thus, the induction of gliosis by the effect of hypoxia was confirmed by this experiment.

### 3.5. Müller Glia Proliferation

**Figure 6:**
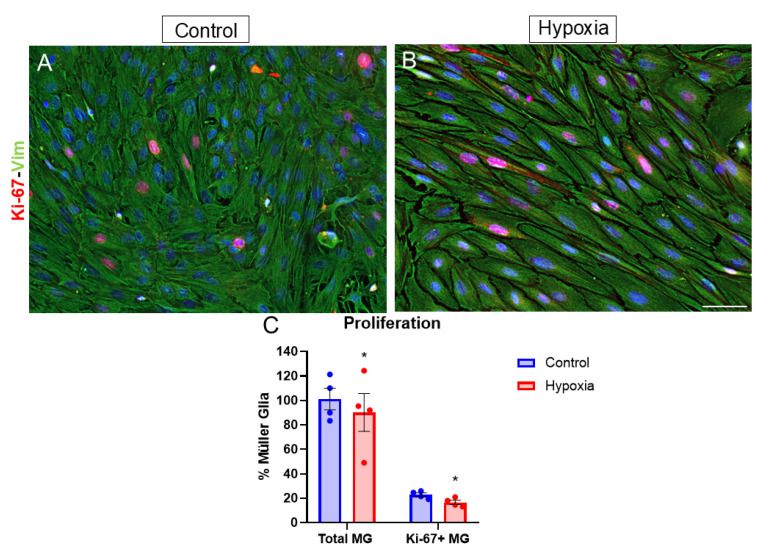
Analysis of MG proliferation through Ki-67 expression. Images of MG cultures taken under control (**A**) and hypoxia (**B**) conditions, in which the expression of Ki-67 (red) and vimentin (green) is observed. A significant decrease in the percentage of cells expressing Ki-67 can be observed under hypoxic conditions compared to the control (**C**). Nuclei were labeled with DAPI (blue). * *p*-value ≤ 0.05. Scale bar = 50 µm.

To study the effect of hypoxia on cell proliferation, as a marker of actively dividing cells, MG cultures were labeled with anti-Ki-67 antibody. The percentage of Ki-67 nuclear-positive cells was quantified (Figure 6A,B). In control cultures, 22.87 ± 1.37% of Müller cells expressed Ki-67, whereas under hypoxic conditions, the percentage decreased significantly (*p*-value ≤ 0.05) to 16.64 ± 1.76% (Figure 6C). In addition, the number of total cells under hypoxic conditions decreased to 90.19% ± 21.82% compared to control conditions (Figure 6C).

### 3.6. Metabolic Stress

**Figure 7:**
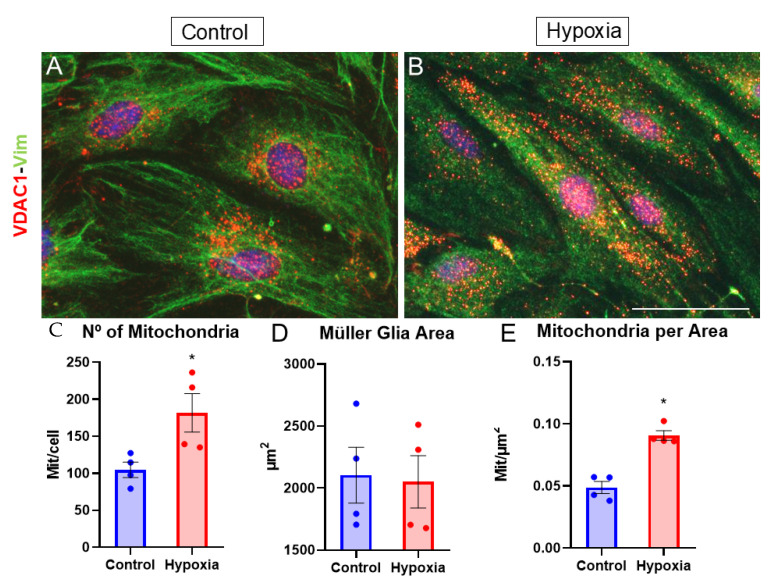
Analysis of mitochondria from MG by VDAC1 labeling. Images of MG cultures taken under control (**A**) and hypoxia (**B**) conditions, in which mitochondria labeled with the anti-VDAC1 antibody (red) and vimentin (green) can be observed. A significant increase in the number of mitochondria can be observed under hypoxic conditions (**C**). A significant increase in the number of mitochondria per µm^2^ cell can also be observed for the same cell area (**D,E**). Therefore, it can be observed that the number of mitochondria labeled with the anti-VDAC1 antibody increases significantly under hypoxic conditions compared to the control. Nuclei were labeled with DAPI (blue). * *p*-value ≤ 0.05. Scale bar = 50 µm.

Due to mitochondria being the source of energy for all cell types, and since they consume a large amount of oxygen, the number of mitochondria in the MG was analyzed by labeling the VDAC1 channel, which is specific to mitochondrial membranes [20]. Under control conditions, it was observed that mitochondria were found mainly around the nucleus (Figure 7A), while under hypoxic conditions, a greater distribution of mitochondria was found throughout the cytoplasm, in addition to around the nucleus (Figure 7B). Likewise, the number of mitochondria/cell (mit/cell) was also quantified (Figure 7C), as well as the number of mitochondria per µm^2^ in both the control and hypoxia samples for the same cell area (Figure 7D). Through this experiment, it was observed that under hypoxic conditions, the number of mitochondria increased significantly (*p*-value ≤ 0.05) to 181.73 ± 26.04 mit/cell compared to the control (104.7 ± 10.45 mit/cell) (Figure 7C). Furthermore, it was also observed that for the same MG area, there was an increase in the number of mitochondria per µm^2^, where in the control it was 0.0488 ± 0.0049 mit/µm^2^, while in the hypoxia sample, it increased significantly (*p*-value ≤ 0.05) to 0.0907 ± 0.0039 mit/µm^2^ (Figure 7D,E), suggesting an increase in metabolic stress when MG are under hypoxic conditions. Furthermore, the mitochondrial size appeared to be influenced by the oxygen conditions, as smaller mitochondria were observed under hypoxic conditions, although this parameter was not quantitatively measured. Notably, under control conditions, the mitochondria were predominantly located around the nucleus. In contrast, hypoxia not only increased the number of mitochondria but also resulted in a wider distribution throughout the cell.

## 4. Discussion

The retina is part of the CNS, and MG are the predominant non-neuronal cell type in the vertebrate retina and account for up to 90% of retinal glia. MG have a wide variety of functions, including maintenance of the blood–retinal barrier, neuroprotection, regulation of the synaptic activity of the different neurons that are found on the different layers of the retina, homeostasis, and the regulation of the cell volume and metabolite reserve [1,2]. Furthermore, the retina has the highest oxygen consumption rate per tissue volume compared of all the organs, including the brain, so its high energy demand makes it a tissue that is highly susceptible to oxygen variations, and therefore to hypoxia [9,10,11]. In different diseases affecting the retina, such as central retinal vein occlusion, glaucoma, diabetic retinopathy, or other diseases related to retinal neovascularization, it has been shown that there is an important hypoxic component [12,13,21].

Although protocols for the in vitro culture of MG exist [13,15,21,22], most rely on neonatal mice or cell lines, which may not fully reflect the conditions in adult mammalian retinas in vivo. Given this limitation, the present study focuses on the effects of hypoxia on adult MG in primary cultures. We examined HIF-1α expression, cell survival, caspase-3-mediated apoptotic cell death, reactive gliosis, cell proliferation, and metabolic stress, indicated by mitochondrial numbers.

We first analyzed the expressions of the MG markers CRALBP, GS, and vimentin, which play key roles in homeostasis and glia–neuron interactions [3], under both control and hypoxic conditions. Besides confirming the purity of our cultures, as these are specific markers for MG, we found no significant differences in their expression. Other studies have reported similar findings [23]. For instance, in a study using the immortalized Müller cell line rMC-1 subjected to hypoxia, CRALBP and GS expressions remained unchanged after 24 h of exposure [22]. In contrast to previous studies reporting an increase in GS levels under hypoxic conditions [15]—likely due to the role of GS in metabolizing excessive extracellular glutamate, which can exert neurotoxic effects on RGCs [2,3]—our results did not show a clear upregulation of GS expression. This discrepancy may be attributable to differences in the age of the rats that were used for MG cell culture: while previous studies used neonatal rats, our experiments employed cells from adult rats. Additionally, the duration of hypoxic exposure may also play a role, as our MG cells were subjected to 72 h of hypoxia, potentially modeling chronic rather than acute hypoxic stress.

When we exposed MG to hypoxia, we observed an increase in HIF-1α expression, validating the in vitro hypoxia model. It is well established that one of the primary cellular responses to hypoxic damage is the upregulation of the transcription factor HIF-1α, which triggers the cell’s adaptive mechanisms to counteract hypoxic injury [12,13]. In a glaucomatous mouse model, immunolabeling of the entire retina and Western blot analysis for HIF-1α protein levels revealed a marked increase in HIF-1α expression, particularly in MG and astrocytes [24]. Numerous studies have confirmed that the HIF-1α expression rises under hypoxic conditions [25,26,27], which aligns with the findings of the present study. However, in neonatal mice, it was reported that HIF-1α is only altered in retinal neurons but not in MG [28]. This discrepancy could be due to the differential behavior of adult MG and neonatal MG cells under hypoxic conditions.

After exposure to hypoxia, a decrease in MG cell survival was observed. Similar findings have been reported in other studies using the methyl-thiazolyltetrazolium (MTT) assay, which measures cell viability and shows reduced levels under hypoxic conditions, indicating decreased survival [29]. This decline in cell survival is linked to increased apoptotic cell death via caspase-3 expression, as a significant rise in caspase-3-positive cells has been noted under hypoxia [30], and decreased proliferation levels have been observed by Ki-67 labeling. Other studies found that under chemically induced hypoxia, such as CoCl_2_ exposure, there were elevated caspase 3 levels [31], which aligns with the findings of this study. A reduction in the proliferative capacity of primary MG cultures under hypoxic conditions was observed, as evidenced by decreased nuclear Ki-67 labeling. These results are consistent with previous studies using the methyl-thiazolyl-tetrazolium (MTT) assay, which reported reduced MG proliferation under hypoxia compared to normoxia [29]. However, contrasting findings have been reported in studies using the rMC-1 Müller glial cell line, where hypoxia was associated with increased proliferation [22]. This discrepancy highlights the differential behavior of primary MG cultures and immortalized MG cell lines under hypoxic conditions.

GFAP, a type III intermediate filament, is the most commonly used marker for studying reactive gliosis in MG [32]. Under pathological conditions such as ischemia/hypoxia, glaucoma, or diabetic retinopathy, the expression of this protein is upregulated, making it a key indicator of damage in retinal glial cells [32,33,34,35]. These results agree with those obtained in this study, as increased GFAP expression can be observed under hypoxic conditions.

Interestingly, we observed that vimentin—also a type III intermediate filament—undergoes structural changes in its organizational pattern under hypoxic conditions. Under control conditions, vimentin mostly showed the parallel, linear organization characteristics of healthy MG cells. Under hypoxia, the vimentin fibers adopted a different reticulated and less uniform organization. This suggests an adaptive reorganization of the vimentin cytoskeleton in response to hypoxia. This phenomenon may occur because vimentin acts as a redox sensor, adopting different configurations in response to various oxidants and electrophiles. As a result, oxidative stress, driven by increased reactive oxygen species (ROS), can alter the structure and organization of vimentin filaments [36]. The changes in the organization of vimentin observed here are likely due to oxidative stress caused by the elevated ROS levels that were generated by mitochondrial metabolic stress under hypoxic conditions.

The retina has the highest rate of oxygen consumption per tissue volume of all the organs [8], making the role of mitochondria indispensable. In cellular metabolism, mitochondria are responsible for using oxygen to produce energy, modulate cellular redox potential, and perform osmotic regulation, pH control, or calcium homeostasis between other [37], and it has been observed that under hypoxia, a downregulation of metabolic activity and ATP production occurs [29,38,39]. Our results indicate that under hypoxic conditions, there is an increase in the number of mitochondria in MG, which translates into increased metabolic stress in these cells. These results have been confirmed by other studies indicating that under conditions of prolonged hypoxia, there is a decrease in mitochondrial autophagy, resulting in an increase in mitochondrial numbers, which leads to an increase in metabolic stress [25].

In addition to reducing autophagy, hypoxia has been shown to induce mitochondrial fission [40,41]. Notably, intrauterine hypoxia also leads to an increase in mitochondrial biogenesis and content, suggesting that hypoxia triggers mitochondrial dysregulation and a reduction in energy metabolism in cortical astrocytes [39]. This dysregulation contributes to increased metabolic stress. These findings provide insights into the observed rise in total mitochondrial numbers and the reduction in mitochondrial size. Furthermore, under hypoxic conditions and elevated HIF-1α expression, complex II of the mitochondrial electron transport chain becomes activated, while the activity of complex I (NADH-ubiquinone oxidoreductase) is downregulated [42,43]. As a result of the reduced complex I activity and limited oxygen availability, NADH accumulates within the mitochondria, disrupting the NADH/NAD^+^ ratio. This imbalance suggests that mitochondrial dysfunction under hypoxic conditions involves not only metabolic stress but also oxidative stress. This may be linked to an increase in the number of mitochondria, as excess NADH could be harmful to MG cells, prompting the need for more mitochondria per cell. Mitochondria adapt dynamically to the energy demands of different cell types and environments. In this study, we observed that oxygen deprivation alters the intracellular distribution of mitochondria, likely linked to increased mitochondrial motility.

Previous research on neurons has shown that the regulator of mitochondrial movement is upregulated under hypoxic conditions, driven by HIF-1α, leading to its redistribution throughout the cytoplasm [44]. Additionally, it has been demonstrated that this redistribution is influenced by the Hypoxia-Upregulated Mitochondrial Movement Regulator (HUMMR), which, under hypoxic conditions, collaborates with HIF-1α to drive anterograde mitochondrial movement from the soma to dendrites and axons in neurons, or from soma to astrocytic projections in astrocytes [45]. In contrast, in cancer cells, mitochondria exhibit a different pattern of localization, often aggregating around the nucleus [40]. However, in the present study, the mitochondria displayed a broader distribution, extending throughout the cytoplasm. These findings underscore the metabolic stress that is experienced by cells under hypoxia, as the redistribution of mitochondria across the cytoplasm is indicative of elevated metabolic demand and stress [44].

## 5. Conclusions

In summary, the retina is a CNS tissue and has the highest metabolic rate in the body, surpassing even the brain, making it particularly vulnerable to fluctuations in oxygen levels. Müller glia (MG), the primary glial cells in the retina, perform numerous functions that are essential for the maintenance of and communication between various retinal cells. Our study reveals that adult MG are highly sensitive to oxygen level changes, with hypoxia leading to reduced proliferation and survival due to apoptosis and increased metabolic stress, which may differ from neonatal MG cultures, making our model better for understanding retinal neurodegenerative diseases. In conclusion, adult MG respond to hypoxia and may play a role in the development of pathologies with a hypoxic component. Further research is necessary to fully understand how these changes impact the retinal physiology and to explore potential strategies to mitigate the effects of hypoxia on adult MG.

## Figures and Tables

**Table 1:** Primary antibodies and the concentrations they were used at.

Antigen	Host	Supplier (Ref)	Concentration
HIF-1α	Mouse	Santa Cruz Biotechnologies (sc-13515)	1:50
Vimentin	Mouse	Dako (M0725)	1:1000
Vimentin	Rabbit	Abcam (ab92547)	1:2000
Glutamine synthetase	Mouse	Abcam (ab64613)	1:1000
CRALBP	Rabbit	Abcam (ab154898)	1:1000
Caspase 3	Rabbit	Cell Signalling (#9661)	1:10,000
GFAP	Mouse	Sigma (G3893)	1:1000
GFAP	Rabbit	Sigma-Aldrich (G9269)	1:1000
Ki-67	Rabbit	Santa Cruz Biotechnologies (sc-23900)	1:200
VDAC1	Rabbit	Abcam (ab15895)	1:200

## Data Availability

The original contributions presented in this study are included in the article/Supplementary Material. Further inquiries can be directed to the corresponding authors.

## Supplementary Materials

The following supporting information can be downloaded at https://www.mdpi.com/article/10.3390/biomedicines13071743/s1. Figure S1. Effect of hypoxia on MG survival. Wide-field images of the entire well of a 24 well plate stained with DAPI and a zoom-in of those images taken of control (A) and hypoxia (B) conditions. It is observed that under hypoxic conditions there is a decrease in cell density. Images of the mosaic taken at 10x.

## Abbreviations

The following abbreviations are used in this manuscript:

ARVO	Association for Research in Vision and Ophthalmology
ATP	Adenosine Triphosphate
BSA	Bovine Serum Albumin
CNS	Central Nervous System
CoCl2	Cobalt(II) Chloride
CRALBP	Cellular Retinaldehyde
DAPI	4′,6-Diamidino-2-Phenylindole
DMEM	Dulbecco’s Modified Eagle’s Medium
EBSS	Earle’s Balanced Salt Solution
FBS	Fetal Bovine Serum
GFAP	Glial Fibrillary Acidic Protein
GLAST	Glutamate Aspartate Transporter
GS	Glutamine Synthetase
HIF	1-Hypoxia-Inducible Factor 1
HIF	1α-Hypoxia-Inducible Factor 1-alpha
HUMMR	Hypoxia-Upregulated Mitochondrial Movement Regulator
Ki67	A Marker of Cellular Proliferation
MG	Müller Glia
MTT	Methyl-Thiazolyl-Tetrazolium
NADH	Nicotinamide Adenine Dinucleotide (reduced form)
PBS	Phosphate-Buffered Saline
RGC	Retinal Ganglion Cell
ROS	Reactive Oxygen Species
SEM	Standard Error of the Mean
SPSS	Statistical Package for the Social Sciences
VDAC1	Voltage-Dependent Anion Channel 1
