# Supplementary figures and images for: Effect of Hypoxia on Adult Müller Glia Cultures
Source: Biomedicines. 2025 Jul 16;13(7):1743. doi: 10.3390/biomedicines13071743 (PMC12292981; doi:10.3390/biomedicines13071743)

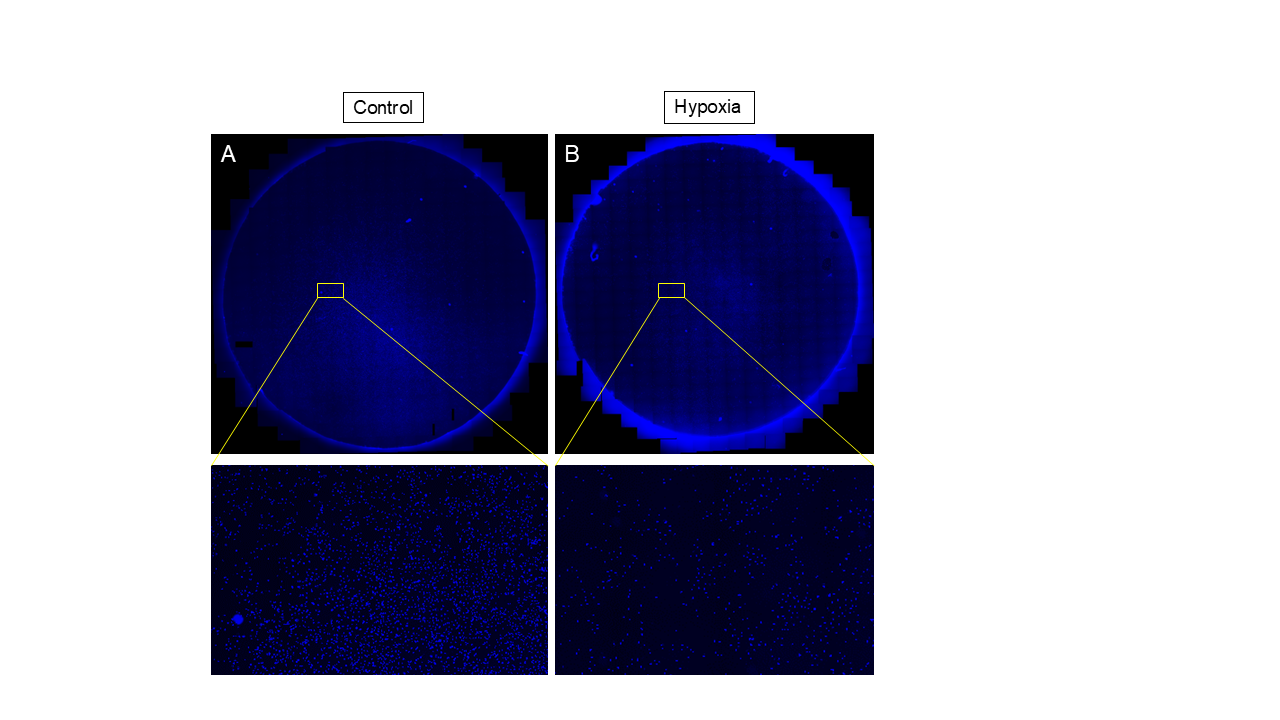

Supplement: Supplementary file 1 [file biomedicines-13-01743-s001.zip › biomedicines-3711889-supplementary.TIF]
